# Clinicopathologic significance of protein lysine methyltransferases in cancer

**DOI:** 10.1186/s13148-020-00897-3

**Published:** 2020-10-13

**Authors:** Theodore Vougiouklakis, Benjamin J. Bernard, Nupur Nigam, Kyunghee Burkitt, Yusuke Nakamura, Vassiliki Saloura

**Affiliations:** 1 Department of Pathology, New York University Langone Health, New York, USA; 2 Thoracic and GI Malignancies Branch, Center for Cancer Research, National Cancer Institute, 41 Medlars Drive, National Cancer Institute, Bethesda, MD 20892 USA; 3 Cancer Precision Medicine Research Center, Japanese Foundation for Cancer Research, Koto, Japan

**Keywords:** Protein lysine methyltransferases, Clinicopathologic associations, Immunohistochemistry

## Abstract

Protein lysine methyltransferases (PKMTs) constitute a large family of approximately 50 chromatin modifiers that mono-, di- and/or tri-methylate lysine residues on histone and non-histone substrates. With the advent of The Cancer Genome Atlas, it became apparent that this family of chromatin modifiers harbors frequent genetic and expression alterations in multiple types of cancer. In this regard, past and ongoing preclinical studies have provided insight into the mechanisms of action of some of these enzymes, laying the ground for the ongoing development of PKMT inhibitors as novel anticancer therapeutics. The purpose of this review is to summarize existing data obtained by different research groups through immunohistochemical analysis of the protein expression levels of PKMTs, and their respective clinicopathologic associations. We focused on studies that used immunohistochemistry to associate protein expression levels of specific PKMTs, as well as several established histone methylation marks, with clinicopathologic features and survival outcomes in various cancer types. We also review ongoing clinical trials of PKMT inhibitors in cancer treatment. This review underscores the clinical relevance and potential of targeting the family of PKMT enzymes as the next generation of cancer therapy.

## Background

It is well recognized that cancer is a genetic disease, though more recently it has become evident that epigenetic aberrations are also involved in critical steps of malignant transformation and progression. Protein lysine methyltransferases (PKMTs) constitute a large family of approximately 50 chromatin modifiers that mono-, di- and/or tri-methylate lysine residues on histone and non-histone substrates. These chromatin modifiers are responsible for the transcriptional regulation of specific downstream target genes, but may also affect the function of non-histone proteins by regulating post-translational modifications, protein-protein interactions, protein stability, and subcompartmental cellular localization of non-histone substrates [1].

The Cancer Genome Atlas has revealed frequent genetic and expression alterations of PKMTs in multiple types of cancer [2, 3]. In this regard, past and ongoing preclinical studies have provided insight into the mechanisms of action of some of these enzymes, laying the ground for the ongoing development of PKMT inhibitors as anticancer therapeutics. While the genetic and expression alterations of PKMTs in various cancer types have been investigated through multiple large scale genomic and transcriptomic studies, and are accessible through a number of publicly available databases, the evaluation of whether these alterations are also reflected at the protein level has not been systematically studied. To this end, multiple efforts are currently ongoing through proteomics approaches.

The purpose of this review is to summarize existing data obtained by different research groups through immunohistochemical analysis of the protein expression levels of PKMTs and their respective clinicopathologic associations in solid tumors. We focused on studies that used immunohistochemistry to associate protein expression levels of specific PKMTs with clinicopathologic features and survival outcomes in various cancer types. Additionally, we also review PKMT inhibitors that are in clinical development. This review underlines the clinical relevance and potential of targeting the family of PKMT enzymes as the next generation of cancer therapy.

## Methods

We used the Pubmed literature database to systematically interrogate and identify original research articles published up to August 2019, investigating protein expression levels of PKMTs using immunohistochemical analysis in clinically annotated tumor samples. We used the search terms “EZH2” or “EHMT2” etc. (other PKMTs, total of 49 enzymes) and “breast cancer” or “non-small cell lung cancer” (other cancer types, as per below). We interrogated each of the PKMTs among some of the most frequent solid cancer types worldwide (breast, non-small cell lung, colorectal, prostate, hepatocellular, gastric, squamous cell carcinoma of the head and neck, endometrial, and ovarian cancer). We included studies that fulfilled the following criteria: (1) protein levels were assessed by immunohistochemistry of tumor samples, which allows for the distinction between cancer and stroma/immune cells, thus ascertaining that the associations derived were cancer-cell specific (we excluded studies of tumor samples by Western blotting for the above reason), (2) the Reporting Recommendations for Tumor Marker Prognostic Studies (REMARK) were followed, (3) samples were clinically annotated and of a sufficient sample size, and (4) contained sufficient data for an estimated hazard ratio relative to overall survival, recurrence-free survival or both, whenever these were assessed. We did not focus on mechanistic studies, unless these included analyses of clinicopathologic associations. Studies that determined overexpression of PKMTs by mRNA quantification (application of polymerase chain reaction or RNA-sequencing) were not included. Applying all the above search criteria, we found eligible literature of 75 articles for the following PKMTs: EZH2 (Enhancer of Zeste Homolog 2), EHMT2 (G9a, Euchromatic Histone Lysine Methyltransferase 2), SMYD2 (SET and MYND Domain Containing 2), SMYD3 (SET and MYND Domain Containing 3), NSD2 (Nuclear Receptor Binding SET Domain Protein 2), NSD3 (Nuclear Receptor Binding SET Domain Protein 3), SETD7 (SET Domain Containing Lysine Methyltransferase 7), SETD1A (SET Domain Containing 1A), SETDB1 (SET Domain Bifurcated 1), and EHMT1 (Euchromatic Histone Lysine Methyltransferase 1).

## Clinicopathologic associations of PKMTs in various cancer types

This section reviews the clinicopathologic associations of PKMTs (EZH2, EHMT2 (G9a), SMYD2, SMYD3, NSD2, NSD3, SETD7, SETD1A, SETDB1, and EHMT1) in various solid cancer types. These data are summarized in Table 1.

**Table 1:** Clinicopathologic associations of PKMTs

**EZH2**		
**Canonical histone modification: H3K27 methylation**		
**Cancer type**	**Correlation with clinicopathologic features**	**Effect on overall survival**
Breast cancer [4–7]	High histologic grade High proliferation index Triple-negative histology Advanced TNM stage Presence of metastasis at diagnosis	Independent predictor of worse disease-specific survival
Non-small cell lung cancer [8–10]	High histologic grade Advanced TNM stage Squamous cell histology	Independent predictor of worse overall survival in lung adenocarcinoma
Colorectal cancer [11–15]	Advanced TNM stage Increased invasion depth	Conflicting studies: • Independent predictor of worse overall survival • Independent predictor of improved overall survival
Endometrial cancer [6, 16, 17]	Aggressive serous papillary and clear cell subtypes High histologic grade High nuclear grade Advanced clinical FIGO stage	Independent predictor of worse overall and progression-free survival
Gastric cancer [18]	No correlation data available	Independent predictor of worse overall and progression-free survival
Prostate cancer [6, 19]	Lymph node metastasis Seminal vesicle invasion Poorly differentiated tumors	Independent predictor of worse recurrence-free and overall survival
Ovarian cancer [20, 21]	Advanced clinical stage High histologic grade	Independent predictor of worse overall survival
Squamous cell carcinoma of the head and neck [22–24]	High histologic grade Advanced clinical stage Tumor stage Lymph node metastasis	Independent predictor of worse overall survival
**EHMT2 (G9a)**		
**Canonical histone modification: H3K9 methylation**		
**Cancer type**	**Correlation with clinicopathologic features**	**Effect on overall survival**
Non-small cell lung cancer [25, 26]	No correlation data available	Independent predictor of worse overall and recurrence-free survival in NSCLC
Gastric cancer [27]	Advanced clinical stage Lymph node metastasis	Independent predictor of worse overall survival
Hepatocellular carcinoma [28]	No correlation data available	Independent predictor of worse overall survival
Ovarian cancer [29]	Advanced clinical stage High histologic grade Serous histology	No association
Squamous cell carcinoma of the head and neck [30]	No correlation data available	Predictor of worse overall survival (no multivariate analysis was conducted to determine if EHMT2 is an independent prognostic factor)
**SMYD2**		
**Canonical histone modification: H3K4/H3K36 methylation**		
**Cancer type**	**Correlation with clinicopathologic features**	**Effect on overall survival**
Gastric cancer [31]	Lymph node metastasis Tumor size Depth of tumor invasion	Independent predictor of worse overall survival
Hepatocellular carcinoma [32]	Vascular invasion Tumor size Advanced clinical stage Poorly differentiated tumors	Independent predictor of worse overall survival
**SMYD3**		
**Canonical histone modification: H3K4/H4K5/H4K20 methylation**		
**Cancer type**	**Correlation with clinicopathologic features**	**Effect on overall survival**
Hepatocellular carcinoma [33]	Tumor size	Independent predictor of worse overall survival
Prostate cancer [34]	No correlation data available	Independent predictor of worse overall survival
**NSD2**		
**Canonical histone modification: H3K36 methylation**		
**Cancer type**	**Correlation with clinicopathologic features**	**Effect on overall survival**
Endometrial cancer [35]	Lymphovascular invasion High histologic grade Lymph node metastasis Depth of myometrial invasion Advanced FIGO clinical stage	Independent predictor of worse overall and progression-free survival
Prostate cancer [36]	No correlation data available	Independent predictor of biochemical recurrence
Squamous cell carcinoma of the head and neck [37]	Poorly differentiated tumors	No correlation data available
**NSD3**		
**Canonical histone modification: H3K36 methylation**		
**Cancer type**	**Correlation with clinicopathologic features**	**Effect on overall survival**
Squamous cell carcinoma of the head and neck [38]	Poorly differentiated tumors	No association
**SETD7**		
**Canonical histone modification: H3K4 methylation**		
**Cancer type**	**Correlation with clinicopathologic features**	**Effect on overall survival**
Breast cancer [39]	Advanced nodal stage	Independent predictor of worse disease-free and overall survival in all breast cancer subtypes
Hepatocellular carcinoma [40]	Tumor size High histologic grade	Independent predictor of worse overall survival
**SETD1A**		
**Canonical histone modification: H3K4 methylation**		
**Cancer type**	**Correlation with clinicopathologic features**	**Effect on overall survival**
Breast cancer [41]	Advanced TNM stage Vascular invasion Metastasis	Independent predictor of worse overall survival in TNBC
**SETDB1**		
**Canonical histone modification: H3K9 methylation**		
**Cancer type**	**Correlation with clinicopathologic features**	**Effect on overall survival**
Colorectal cancer [42, 43]	High histologic grade Advanced TNM stage	Independent predictor of worse overall survival
Hepatocellular carcinoma [44, 45]	Progressively increasing SETDB1 protein levels from normal liver to chronic hepatitis to HCC	No correlation data available
**EHMT1**		
**Canonical histone modification: H3K9 methylation**		
**Cancer type**	**Correlation with clinicopathologic features**	**Effect on overall survival**
Gastric cancer [46]	Tumor stage Lymph node metastasis	No correlation data available

Higher expression levels of the above PKMTs are associated with the above described adverse clinicopathologic features unless stated otherwise

### EZH2 (Enhancer of Zeste Homolog 2)

Clinicopathologic associations of EZH2, a transcriptional repressor that induces H3K27 tri-methylation (H3K27me3), have been drawn in many cancer types, including breast cancer, non-small cell lung cancer (NSCLC), colorectal cancer, endometrial cancer, gastric cancer, prostate cancer, ovarian cancer, and squamous cell carcinoma of the head and neck (SCCHN).

#### Breast cancer

Immunohistochemical analysis of EZH2 in breast cancer supports that higher expression levels of EZH2 correlate with aggressive features and poor prognosis. Guo et al. conducted immunohistochemical analysis of EZH2 in 226 breast cancer tissues of various subtypes, and found EZH2 overexpression in 61% of cases [4]. EZH2 expression correlated significantly with high-grade ductal carcinoma in situ, triple negative breast cancer (TNBC), Ki-67 proliferation index, and HER2 positivity [4]. The highest EZH2 protein expression levels were observed in TNBC, while the lowest expression levels were found in estrogen receptor (ER)/progesterone receptor (PR)-positive breast cancers with low proliferative index. This finding supports the aggressive nature of TNBCs in contrast to hormone receptor positive breast cancers. Associations with established risk factors such as clinical stage and lymph node status have been shown in highly expressing EZH2 breast cancers. A study comprised of 194 cases with available clinical follow-up revealed that higher EZH2 protein levels were linked with shorter disease-specific survival in lymph node-negative and stage I and II disease, however not in lymph node-positive and stage III and IV disease [5]. These associations were independent of ER status. Overall, the 10-year disease-free survival in patients with high EZH2 expression was significantly lower at 24.7% when compared to patients with low EZH2 expression at 58.9%. EZH2 expression was an independent predictor of disease-specific survival after multivariate analysis (explanation of univariate/multivariate analysis in Explanation of staging and statistical terms section) including PR expression and positive lymph node status [5]. Similar findings were reported by a subsequent study of 190 breast cancer cases, with high EZH2 protein expression associated with higher histologic grade, locally advanced cancers and the presence of metastatic disease at the time of diagnosis [6]. Moreover, additional investigators have found that higher EZH2 levels are independently associated with worse 5-year overall and disease-free survival in a large series of 410 breast cancer cases [7]. These studies suggest that EZH2 is an independent prognostic factor of poor survival in breast cancer patients.

#### Non-small cell lung cancer

The potential oncogenic role of EZH2 and its clinical relevance has also been investigated in NSCLC. Results from a small cohort of 69 surgically resected lung adenocarcinomas revealed EZH2 immunopositivity in 63.8% of tumor tissues, and correlation with higher histologic grade and advanced TNM stage, suggesting that EZH2 may contribute to malignant disease progression [8]. In an additional series consisting of 63 squamous cell carcinomas and 82 adenocarcinomas, significantly higher EZH2 expression was seen in squamous cell carcinomas in relation to adenocarcinomas [9]. High EZH2 expression was present in 62% of all NSCLCs and its expression was positively associated with non-adenocarcinoma histology, higher tumor stage (T2-T4), histologic grade and proliferation index. While higher EZH2 expression rendered poorer prognosis when compared to low EZH2 expressing tumors across all NSCLC stages, in multivariate analysis, EZH2 was an independent prognostic factor of overall survival only in stage I NSCLC patients [9].

Behrens et al. comprehensively characterized EZH2 protein expression in 541 primary NSCLC tumors comprised of 221 squamous cell carcinomas and 320 adenocarcinomas [10]. In their series, hyperplastic epithelium, low-grade, and high-grade dysplastic lesions displayed gradually increasing EZH2 expression levels when compared to normal bronchial epithelium [10]. Higher EZH2 expression levels correlated with younger age, smoking history and advanced TNM stage (explanation of TNM staging provided in Explanation of staging and statistical terms section) in adenocarcinomas, and importantly, rendered worse recurrence-free and overall survival independently of other known prognostic factors, such as TNM stage, tumor size and adjuvant therapy [10]. Furthermore, *KRAS* Gly to Cys substituted NSCLC tumors had significantly higher EZH2 expression levels, however no other associations with mutated *EGFR* or other *KRAS* mutations were found. Although squamous cell lung carcinomas had increased EZH2 expression levels compared to lung adenocarcinomas, the only significant association observed with increased EZH2 expression was lymph node metastasis [10]. These findings support that EZH2 is overexpressed in aggressive NSCLCs and may merit further investigation as a potential novel therapeutic target in NSCLC.

#### Colorectal cancer

Immunohistochemical interrogation of EZH2 has demonstrated correlation between high expression levels, adverse clinicopathologic features and worse survival in patients with colorectal cancer. In a series of 119 colorectal cancer tissues and their adjacent normal counterparts, immunohistochemical analysis of EZH2 showed that 69.7% of cases overexpressed EZH2, and overexpression was associated with advanced TNM stage and increased depth of invasion [11]. Importantly, this study also found that EZH2 was an independent predictor of decreased overall survival in patients with colorectal cancer after multivariate analysis for lymph node and distant metastasis. These findings were supported by another study of 95 patients with colorectal cancer, in which high protein levels of EZH2 were associated with advanced clinical stage, histologic grade and independently predicted poor overall survival in this patient cohort [12]. Furthermore, Ohuchi et al. showed that EZH2 protein expression levels gradually increased from normal colonic mucosa to dysplasia and colorectal cancer, suggesting an oncogenic role for EZH2 in the carcinogenesis process from benign mucosa to carcinoma [13].

Conversely, a recent meta-analysis of 8 studies comprised of 1059 patients with colorectal cancer concluded that higher EZH2 expression evaluated by immunohistochemistry was associated with significantly improved overall survival [HR = 0.58, CI 95% (0.38–0.79)], suggesting that in patients with colorectal cancer, EZH2 may function as a tumor suppressor [14]. This contradicts the aforementioned studies, which show that high EZH2 expression is associated with poor survival in colorectal cancer patients, as well as many other cancer types as reviewed in this article. One important point of concern in this meta-analysis is that there was no multivariate analysis conducted to assess EZH2 as an independent prognostic factor for colorectal cancer. As such, other established factors of prognostic significance, such as clinical stage and histologic grade, could be responsible for the reported favorable survival impact. Corroborating this point, detailed clinical characteristics of the patients included in the analysis were not provided. As the authors also point out, other limitations of this meta-analysis include differences in the duration of follow-up, different immunohistochemistry protocols and assessment methods of EZH2 protein expression levels and the absence of a standardized cut-off defining decreased or increased EZH2 expression. Regardless, recent studies suggest that EZH2 is a dual-faced molecule that may function as a transcriptional repressor or activator depending on the cell context, which may dictate variable post-translational modifications and variations in the interactions between EZH2 and other Polycomb Repressive Complex 2 (PRC2) subunits [15]. It could be postulated that such biologic differences could be responsible for “switches” of function of EZH2 not only among different cancer types, but potentially also among different stages of the same cancer type. In summary, the above underline the need for further rigorous preclinical investigation and prognostic assessment of EZH2 before it is pursued as an anticancer drug target in colorectal cancer.

#### Endometrial cancer

EZH2 has been identified as a critical PKMT that correlates with multiple adverse clinicopathologic parameters in endometrial cancer. Bachmann et al. examined EZH2 protein expression levels in a series of 316 endometrial tumor samples and found that higher EZH2 expression correlated with the aggressive serous papillary and clear cell carcinoma subtypes, high histologic and nuclear grade, and advanced clinical FIGO stage (International Federation of Gynecology and Obstetrics, explanation of FIGO staging in Explanation of staging and statistical terms section) [6]. Furthermore, the 5-year survival rates of low and highly EZH2 expressing endometrial cancers were 80% and 56% respectively, and were independent of other known prognostic factors such as histologic type, grade, vascular invasion, depth of myometrial infiltration, and clinical FIGO stage. A subsequent study by Zhou and colleagues investigated EZH2 protein expression levels in 202 cases comprised of type-1 (endometrioid adenocarcinomas, *n* = 141) and type-2 (serous and clear cell carcinomas, *n* = 61) endometrial cancers, and detected overexpression in 7.6% and 63% of tumors respectively [16]. EZH2 overexpression correlated significantly with high histologic grade, lymphovascular and myometrial invasion, lymph node metastasis and the clinically aggressive type-2 tumors. This study further identified EZH2 as an independent dismal predictor for overall survival, after accounting for known prognostic factors, such as age, race, tumor type, lymphovascular invasion, tumor grade, and clinical FIGO stage [16]. Another series of 104 endometrial cancer cases revealed that higher EZH2 protein expression was independently and significantly correlated with worse progression-free survival, but not with overall survival [17]. Findings from the above studies support that EZH2 functions as an oncogene and merits further preclinical investigation as a novel drug target in endometrial cancer.

#### Gastric cancer

Studies involving the immunohistochemical evaluation of EZH2 expression in gastric cancer have revealed that EZH2 significantly correlates with poor prognosis. In a cohort of 117 gastric cancer cases with corresponding normal tissues, 70.1% of tumor samples were positive for EZH2 when compared to 5.4% of benign gastric mucosa samples [18]. Similarly, 56.4% of cancer tissues were positive for H3K27me3, the enzyme end-product of EZH2, compared to 7.3% of normal gastric mucosa samples. Importantly, overexpression of EZH2 or H3K27me3 protein levels were found to be independent predictors of overall and progression-free survival after multivariate analysis for tumor size, histologic grade and clinical stage [18]. More specifically, high EZH2 and H3K27me3 expression correlated with median overall survival of 25.2 and 23.4 months respectively [18]. Conversely, low EZH2 and H3K27me3 expression correlated with median overall survival of 40.5 and 37.6 months respectively. Interestingly, when the expression levels of both EZH2 and H3K27me3 were considered together, the median overall survival was 18.8 months in high expressors, compared to 43.9 months in low expressors. These findings underscore the potential prognostic impact of EZH2 in patients with gastric cancer.

#### Prostate cancer

Studies have shown associations between EZH2 and poor prognosis in prostate cancer. In a study of 104 prostate cancer patients, high EZH2 expression was associated with lymph node involvement, seminal vesicle invasion, and moderately or poorly differentiated grade [6]. The 10-year survival rate was 93% and 53% in cases with low and high EZH2 expression respectively, and EZH2 was an independent predictor of recurrence-free and overall survival in multivariate analysis. In a separate study comprised of 64 cases, EZH2 protein expression levels independently predicted worse recurrence-free survival and outperformed other known prognostic factors, such as Gleason score, tumor size and preoperative prostate-specific antigen (PSA) levels [19]. Furthermore, significantly higher EZH2 protein levels were observed in metastatic prostate cancer tumor samples compared to localized disease. These findings highlight the function of EZH2 in prostate cancer, and the possibility of EZH2 as a therapeutic target in this disease.

#### Ovarian cancer

EZH2 overexpression has been reported in ovarian cancer, predominantly in the context of epithelial ovarian neoplasms. Rao et al. examined EZH2 expression patterns in 179 ovarian carcinomas and detected positive EZH2 staining in 49.7% of cases, while no immunoreactivity was seen in normal ovarian tissue [20]. The histologic subtypes investigated were primarily serous and mucinous carcinomas, and in smaller percentage clear cell, endometrioid and undifferentiated carcinomas. Clinicopathologic variables associated with EZH2 expression included TNM stage, histologic grade and FIGO stage, while multivariate analysis revealed EZH2 as an independent prognostic factor for dismal overall survival. Concordantly, Li and colleagues detected high EZH2 expression in 66% of 134 epithelial ovarian cancer cases and absent immunoreactivity in normal ovarian tissues [21]. In this cohort, EZH2 correlated with histologic grade and Ki-67 proliferation index, however no significant association was observed with overall or disease-free survival. These studies and the little or no expression of EZH2 in normal ovarian tissues underscore that EZH2 could be an effective therapeutic target in ovarian cancer.

#### Squamous cell carcinoma of the head and neck

EZH2 has also been shown to correlate with clinicopathologic features and survival outcome in patients with SCCHN. Kidani et al. showed that EZH2 protein expression levels were significantly increased in carcinomas compared to dysplasia and normal squamous epithelium, and EZH2 correlated with clinical stage, lymph node metastasis and high histologic grade [22]. While survival analysis in a cohort of 102 patients with oral SCCHN showed that high EZH2 levels predicted worse overall survival, no multivariate analysis was conducted to decipher its independence as a prognostic factor. In another study of 46 patients with locoregionally advanced SCCHN, EZH2 protein overexpression was determined as an independent predictor of overall survival and outperformed lymph node metastasis, a known prognostic factor in SCCHN [23]. Higher EZH2 expression levels also correlated with high histologic grade, whereas no associations with human papillomavirus (HPV)-status were found. Concordantly, Wang et al. examined protein expression levels of EZH2 in 67 patients with SCCHN and found significant correlations with tumor stage, lymph node metastasis and clinical stage, while multivariate analysis identified EZH2 as an independent predictor of overall survival [24]. These studies establish a connection between EZH2 and SCCHN, with multiple studies showing that EZH2 is an independent prognostic predictor in SCCHN.

### EHMT2 (G9a) (Euchromatic Histone Lysine Methyltransferase 2)

Clinicopathologic associations of EHMT2, a transcriptional repressor that methylates H3K9 and H3K27, have been reported in NSCLC, gastric cancer, hepatocellular carcinoma (HCC), ovarian cancer, and SCCHN.

#### Non-small cell lung cancer

Increased EHMT2 protein expression has been reported in lung cancer tissues, including adenocarcinomas and squamous cell carcinomas, when compared to normal control tissues [25, 26]. Chen et al. analyzed EHMT2 protein expression levels in 160 NSCLC samples and demonstrated that patients with high EHMT2 expression had reduced overall and disease-free survival [25]. Furthermore, they showed that EHMT2 promoted the invasion and metastatic potential of lung cancer cells through H3K9 di-methylation (H3K9me2) and silencing of the promoter of *EPCAM*, a cell adhesion molecule. These findings support that EHMT2 warrants further exploration as a drug target in NSCLC [25].

#### Gastric cancer

Overexpression of EHMT2 is also encountered in gastric cancer and has been shown to correlate with aggressive pathologic features. More specifically, a study of 107 gastric cancer tissues with their normal counterparts found that EHMT2 is significantly overexpressed in gastric cancer tissues and correlates with advanced clinical stage and lymph node metastasis [27]. In addition, multivariate analysis showed that patients with high tumor EHMT2 protein expression had significantly worse 5-year overall survival compared to patients with low tumor EHMT2 expression, and this was independent of other established prognostic factors [27]. While further investigation into the role of EHMT2 in gastric cancer is warranted, these findings highlight important prognostic associations.

#### Hepatocellular carcinoma

Additional studies have implicated EHMT2 in HCC pathogenesis. EHMT2 protein overexpression was investigated in a cohort of 350 HCC patients and was independently associated with worse overall survival [28]. While this study links EHMT2 with survival data in HCC, further investigation into the relationship of EHMT2 and HCC is warranted.

#### Ovarian cancer

EHMT2 has been reported to contribute to tumor progression and metastasis in ovarian cancer. Hua et al. showed aberrant EHMT2 protein expression in a series of 208 epithelial ovarian carcinomas with immunopositivity detected in 71.6% of tumors [29]. Analysis revealed that EHMT2 overexpression on immunohistochemistry correlated with advanced FIGO stage, higher histologic grade, and serous type carcinomas. Univariate analysis supported that EHMT2 was associated with overall survival, however after multivariate analysis, this association was lost. Furthermore, their study identified that peritoneal, omental, and nodal metastases displayed increased EHMT2 immunoexpression relative to corresponding primary tumors [29]. These data suggest that EHMT2 may contribute to metastasis, a crucial factor for mortality in these patients.

#### Squamous cell carcinoma of the head and neck

EHMT2 overexpression has been reported in SCCHN. Li et al. examined the protein levels of EHMT2 in a cohort of 108 patients with SCCHN and found significant overexpression in cancer tissues compared to normal squamous epithelium [30]. In a separate cohort of 77 patients, higher EHMT2 protein expression levels predicted worse overall survival, however no multivariate analysis was conducted to assess whether EHMT2 serves as an independent prognostic factor [30]. These findings demonstrate that EHMT2 plays a significant role in SCCHN, potentially serving as a therapeutic target with accrual of more research.

### SMYD2 (SET and MYND Domain Containing 2)

Clinicopathologic associations of SMYD2, known to methylate H3K4 and H3K36, have been reported in gastric cancer and HCC.

#### Gastric cancer

SMYD2 has also been found to have a negative impact on gastric cancer survival. SMYD2 protein expression levels were determined by immunohistochemistry in a cohort of 147 primary gastric cancer samples and its overexpression significantly correlated with features that influence patient survival, such as lymph node metastasis, larger tumor size and depth of tumor invasion [31]. Furthermore, patients with higher SMYD2 expression had significantly worse overall survival, which remained significant after multivariate analysis. This study highlights the negative prognostic impact of SMYD2 in gastric cancer.

#### Hepatocellular carcinoma

SMYD2 is aberrantly expressed in HCC and represents an independent biomarker for dismal prognosis. In a large series comprised of 163 primary HCC cases, 74.8% stained positive for SMYD2 using immunohistochemistry, and tumors displaying SMYD2 immunopositivity were significantly associated with vascular invasion, larger tumor size, higher TNM stage and moderate to poorly differentiated phenotypes [32]. Univariate analysis demonstrated that SMYD2 overexpression conferred poor overall survival, and multivariate analysis confirmed SMYD2 as an independent predictor of overall survival in HCC patients. Based on these findings, SMYD2 may play a significant role in HCC, and further investigation into relevant functions is necessary.

### SMYD3 (SET and MYND Domain Containing 3)

Clinicopathologic associations of SMYD3, which is known to methylate H3K4 and H4K20, have been investigated in HCC and prostate cancer.

#### Hepatocellular carcinoma

Correlative features have been encountered in HCC with SMYD3 that support its oncogenic role and potential utility to predict adverse clinical outcome. SMYD3 protein expression levels appear to convey an unfavorable prognosis in HCC. In a cohort of 100 HCC cases, Fei et al. showed that high SMYD3 expression correlated with HCC tumor size and poor overall survival [33]. In this study, patients with low expressing SMYD3 tumors had a 5-year overall survival rate of 48.7%, in contrast to the highly expressing SMYD3 group of 29.6%. Applied multivariate analysis further revealed high SMYD3 expression as an independent marker of prognosis [33]. These data support that SMYD3 may be an important target in HCC.

#### Prostate cancer

While further investigation into the function of SMYD3 in prostate cancer is necessary, SMYD3 overexpression was associated with worse disease-specific survival in a cohort of 189 prostate cancer patients independently of other known prognostic factors [34].

### NSD2 (Nuclear Receptor Binding SET Domain Protein 2)

Clinicopathologic associations of NSD2, known to di- and tri-methylate H3K36 (H3K36me2, H3K36me3), have been reported in endometrial cancer, prostate cancer, and SCCHN.

#### Endometrial cancer

Studies have shown that NSD2 is overexpressed in endometrial cancer compared to normal endometrium and is associated with adverse prognostic features. More specifically, Xiao et al. examined NSD2 protein expression by immunohistochemistry in 161 endometrial cancers and 62 normal endometrium samples, and detected nuclear positivity in 46.6% and 1.6% respectively [35]. NSD2 overexpression correlated with known prognostic factors, such as lymphovascular invasion, high histologic grade, lymph node metastasis, depth of myometrial invasion and higher FIGO clinical stage. NSD2 expression was also found to be an independent factor for poor overall and disease-free survival in multivariate analysis [35]. These findings suggest that NSD2 could serve as a potential therapeutic target in endometrial cancer.

#### Prostate cancer

High NSD2 protein expression determined by immunohistochemistry has been reported as an independent predictor of biochemical recurrence in a cohort of 108 patients with prostate cancer following radical prostatectomy [36]. Further investigation into the role of NSD2 in prostate cancer is warranted.

#### Squamous cell carcinoma of the head and neck

Significantly higher expression levels of NSD2 have been reported in SCCHN when compared to normal or dysplastic tissues, suggesting that NSD2 may be important in the initial stages of SCCHN oncogenesis. In a study of patients with locoregionally advanced SCCHN, NSD2 was overexpressed in 73% of 149 SCCHN cases [37]. Moreover, high NSD2 levels correlated with poor differentiation. Further investigation of NSD2 and its association with tumor, lymph node or clinical stage, and survival outcomes will be critical as these have not been studied in the literature.

### NSD3 (Nuclear Receptor Binding SET Domain Protein 3)

Clinical associations of NSD3, a H3K36 methylator, have been reported in SCCHN.

#### Squamous cell carcinoma of the head and neck

NSD3 has been studied in SCCHN and has been found to possess oncogenic properties via transcriptionally mediated pathways and direct methylation of proteins. NSD3 is significantly overexpressed in SCCHN compared to normal or dysplastic epithelium [38]. In a study of patients with locoregionally advanced SCCHN, NSD3 was overexpressed in 58% of 132 SCCHN cases [38]. Moreover, high NSD3 expression correlated with poor differentiation and heavy smoking status. No associations though were found with tumor, clinical stage and survival outcomes. These findings suggest that NSD3 may be important in the initial stages of SCCHN oncogenesis.

### SETD7 (SET Domain Containing Lysine Methyltransferase 7)

Clinicopathologic associations of SETD7, a PKMT that mono-methylates H3K4 (H3K4me1) and activates transcription, have been reported in breast cancer and HCC.

#### Breast cancer

Upregulation of SETD7 has also been correlated with advanced nodal stage, tumor size and poor outcome in a cohort of 80 patients with all four molecular subtypes of breast cancer [39]. In this study, multivariate analysis identified high SETD7 protein levels as a significant, independent prognostic indicator of worse disease-free and overall survival. While further investigation of the role of SETD7 in breast cancer is warranted, these findings demonstrate that SETD7 could be a potential therapeutic target in breast cancer.

#### Hepatocellular carcinoma

Significant associations have been reported between SETD7 expression and clinicopathologic parameters in HCC. Immunohistochemical analysis of 225 primary HCC tumors demonstrated SETD7 overexpression compared to neighboring non-tumorous counterparts and correlation with adverse clinicopathologic features, such as tumor size and histologic grade [40]. High SETD7 expression also rendered poorer overall survival on Kaplan-Meier analysis and was identified as an independent prognostic indicator. These findings highlight the potential function of SETD7 in HCC pathogenesis and point to the possibility of targeting SETD7 in HCC therapeutics.

### SETD1A (SET Domain Containing 1A)

Clinicopathologic associations of SETD1A, a H3K4 methylator known to function as a transcriptional activator, have been reported in breast cancer.

#### Breast cancer

Zhu et al. detected SETD1A immunopositivity in 50.9% of 159 TNBCs with 5-year overall survival rates at 3.5% in relation to 39.6% in SETD1A-negative tumors [41]. This association persisted after multivariate analyses for known independent predictors of survival in breast cancer, such as TNM stage and histologic grade were performed. Additionally, higher SETD1A levels were associated with more advanced clinical stage, vascular invasion and metastasis [41]. These findings underpin the role of SETD1A in breast cancer pathogenesis and its potential therapeutic impact in this disease.

### SETDB1 (SET Domain Bifurcated 1)

Clinicopathologic associations of SETDB1, a methylator of H3K9 that induces transcriptional repression, have been reported in colorectal cancer and HCC.

#### Colorectal cancer

Overexpression of SETDB1 has been reported to correlate with aggressive features in colorectal cancer and is linked with worse survival [42, 43]. SETDB1 protein expression levels were significantly associated with advanced TNM stage and high histologic grade in colon adenocarcinomas. Additionally, high SETDB1 expression levels conferred poor overall survival independently of other known prognostic factors, such as TNM stage, in a cohort of 90 colon cancer patients [42]. In line with these findings, additional studies have found that increased SETDB1 protein expression levels correlate with poor prognosis in colorectal cancers [43]. Immunohistochemical analysis of 102 colorectal adenocarcinoma cases revealed that highly expressing SETDB1 cancers rendered a lower 5-year overall survival rate at 35.3%, compared to 76.6% in low SETDB1 expressing cancers [43]. Mechanistically, SETDB1 promoted the proliferation and migratory potential of colorectal cancer cells, and bound and silenced the promoter of *TP53*, attenuating the apoptosis-inducing effect of 5-fluorouracil [43]. These findings demonstrate the potential role of SETDB1 in colorectal cancer pathogenesis.

#### Hepatocellular carcinoma

SETDB1 was found to be overexpressed in a study of 59 HCC tumors compared to normal tissue counterparts [44]. Overexpression of SETDB1 by immunohistochemical evaluation was also reported in another cohort of 89 HCC tumor tissues [45]. Interestingly, in this study the authors found that the median SETDB1 protein levels increase progressively along the continuum from normal liver to chronic hepatitis to HCC, suggesting a role for SETDB1 in the oncogenesis of HCC. Survival and prognostic analyses are warranted to further assess the impact of SETDB1 on the survival of patients with HCC.

### EHMT1 (Euchromatic Histone Lysine Methyltransferase 1)

Clinicopathologic associations of EHMT1, a transcriptional repressor that functions via H3K9 methylation, have been reported in gastric cancer.

#### Gastric cancer

EHMT1 was found to be significantly overexpressed in 97 gastric cancer tissues compared to their normal counterparts, and was associated with tumor stage and lymph node metastasis [46]. No associations, however, with overall survival were available for EHMT1. While further investigations are warranted, these preliminary findings demonstrate that EHMT1 may play a significant role in gastric cancer pathogenesis.

## Clinicopathologic associations of specific histone methylation marks in various cancer types

In the second part of this review, we focus on clinicopathologic and prognostic data of various histone methylation marks in cancer. While some members of the PKMT family have been reported to methylate non-histone substrates, the majority of PKMTs are known to methylate histone substrates. It is well described that histone modifications play key roles in the epigenetic regulation of cancer cells, including implications in carcinogenic mechanisms. As such, they may potentially be associated with important clinicopathologic and prognostic features. In this section, we will highlight key histone methylation marks and some of their reported clinicopathologic associations and prognostic impact. Additionally, Table 2 summarizes the associations between various histone methylation marks (H3K4me2/me3, H3K27me3, H3K36me3, H3K9me3) and clinicopathologic parameters in various cancer types.

**Table 2:** Associations between histone methylation marks and clinicopathologic parameters in various cancer types

**H3K4me2**	
Cancer type	Clinicopathologic association
Non-small cell lung cancer [47, 48] Pancreatic adenocarcinoma [47] Colorectal cancer [47] Esophageal squamous cell carcinoma [47] Hepatocellular carcinoma [47] Cervical cancer [47] Prostate cancer [49] Renal cell carcinoma [48]	Lower **H3K4me2** correlates with worse survival
**H3K4me3**	
Cancer type	Clinicopathologic association
Non-small cell lung cancer [47] Pancreatic adenocarcinoma [47] Colorectal cancer [47, 50] Esophageal squamous cell carcinoma [47] Hepatocellular carcinoma [47, 51] Cervical cancer [47]	Higher **H3K4me3** correlates with worse survival
**H3K27me3**	
Cancer type	Clinicopathologic association
Colorectal cancer [52]	Higher **H3K27me3** correlates with poor survival
Hepatocellular carcinoma [53]	Higher **H3K27me3** correlates with larger tumor size, poorly differentiated tumors, advanced clinical stage, vascular invasion, and poor survival
Esophageal squamous cell carcinoma [54, 55]	Higher **H3K27me3** correlates with advanced TNM stage and poor survival
Gastric cancer [56]	Higher **H3K27me3** correlates with poorly differentiated tumors, distant metastasis and poor survival
Breast cancer [57, 58]	Higher **H3K27me3** correlates with lower tumor grade and better survival
Non-small cell lung cancer [59]	Higher **H3K27me3** correlates with better survival
**H3K36me3**	
Cancer type	Clinicopathologic association
Hepatocellular carcinoma [60]	Higher **H3K36me3** correlates with higher tumor grade, advanced TNM stage and worse survival
Renal cell carcinoma [61]	Lower **H3K36me3** correlates with worse survival
**H3K9me3**	
Cancer type	Clinicopathologic association
Colorectal cancer [62]	Higher **H3K9me3** correlates with lymph node metastasis
Gastric cancer [56, 63]	Higher **H3K9me3** correlates with advanced tumor stage, lymphovascular invasion and worse survival
Non-small cell lung cancer [64]	Higher **H3K9me3** correlates with tumor recurrence
Esophageal squamous cell carcinoma [65]	Higher **H3K9me3** correlates with worse survival

### H3K4 methylation

H3K4 methylation is a key histone modification. H3K4 refers to lysine 4 from the N-terminus of histone H3. H3K4 can be mono-methylated (H3K4me1), di-methylated (H3K4me2), or tri-methylated (H3K4me3), with each unique mark having its own specific phenotypic effect [49]. Due to a growing amount of literature surrounding H3K4 methylation and the effect of this imprint in cancer, Li et al. conducted a meta-analysis to analyze the association between H3K4 methylation (specifically H3K4me2 and H3K4me3) and survival in NSCLC, pancreatic, colorectal, esophageal squamous cell carcinoma (ESCC), HCC, and cervical cancers [47]. In their meta-analysis of 1474 cases of malignant tumors, they found that decreased H3K4me2 expression significantly correlated with poor overall survival, while decreased H3K4me3 significantly correlated with improved overall survival [47]. Furthermore, Seligson et al. first demonstrated that prostate cancer patients with lower expression levels of H3K4me2 (in addition to H3K18ac) had poorer prognosis with an increased rate of tumor recurrence when compared to patients with higher expression levels of this modification [49]. They then further expanded on these findings and demonstrated that lower levels of H3K4me2 and H3K18ac also confer poorer prognosis in renal cell carcinoma and lung adenocarcinoma [48]. In another study, Benard et al. demonstrated that H3K4me3, H3K9me3, and H4K20me3 all possess prognostic value in early-stage colon cancer; more specifically high expression of H3K4me3, and low expression of H3K9me3 and H4K20me3 were correlated with shorter patient survival and higher chances of tumor recurrence [50]. Interestingly, the combined favorable expression profile of low H3K4me3, high H3K9me3, and high H4K20me3 was associated with the best prognosis in regards to patient survival and tumor recurrence when compared to any of the methylation marks independently [50]. Further, He et al. demonstrated that increased expression of H3K4me3 is not only correlated with, but is also an independent predictor of poor survival in patients with HCC, especially in the early stages (TNM I/II) [51]. While just a brief overview of the evidence surrounding H3K4 marks, these data highlight the clinicopathologic importance of H3K4 methylation marks in various cancer types.

### H3K27me3

H3K27 represents lysine 27 of histone H3 and its modification regulates critical pathways in normal and cancer cells. EZH2 mediates the addition of three methyl groups on H3K27 (H3K27me3). The primary known function of H3K27me3 is silencing of gene expression. Regarding its clinicopathologic relevance, the link between H3K27me3 and cancer prognosis is still open-ended. In this section, we focus on studies that present a correlation between H3K27me3 and cancer prognosis.

In a study conducted on 119 primary colorectal cancer tissues, Fornaro et al. reported significantly higher H3K27me3 expression levels in patients with worse prognosis as compared to those with better prognosis [52]. In two independent cohorts of HCC samples and their corresponding normal counterparts, Cai et al. reported high H3K27me3 expression in 63.2% and 60.4% of samples, which significantly correlated with larger tumor size, poor differentiation, advanced clinical stage, vascular invasion, and shorter patient survival [53]. In ESCC, studies reported strong expression of H3K27me3 in the nucleus of cancer cells, which correlated with advanced T and N stage and poor overall survival [54, 55]. Li et al. showed a similar trend in 133 gastric cancer tumor samples, where expression levels of H3K9me2, H3K9me3 and H3K27me3 were examined and elevated levels of all three histone marks were found in cancer tissues compared to peri-cancer tissues [56]. H3K27me3 expression was associated with the degree of tumor differentiation and also correlated with distant metastasis in patients with gastric cancer. Patients with high levels of H3K27me3, H3K9me2, and H3K9me3 exhibited a lower overall survival rate [56].

Interestingly, while the above studies link high H3K27me3 with poor prognosis, there are studies that display an inverse correlation. In breast cancer tissues, higher levels of H3K27me3 were associated with lower tumor grade and better survival [57, 58]. In the same tissues, high expression of EZH2, which tri-methylates H3K27, was also associated with poor survival, as previously reported [4]; though one would expect high expression levels of EZH2 to correlate with high expression levels of H3K27me3. H3K27me3 expression was lowest in more aggressive subtypes of breast cancer, specifically the basal-like, triple negative, luminal B, and ER-positive tumors with high proliferation index, similar to EZH2 which was highest in basal-like and triple negative tumors [57]. A similar relationship was reported by Chen et al. in a cohort of 42 NSCLC tumor tissues, where higher H3K27me3 expression levels correlated with lower tumor invasiveness and better disease-free survival [59]. In comparison to normal lung tissue, H3K27me3 expression levels were decreased, while EZH2 expression levels were increased in lung adenocarcinoma and squamous cell carcinoma tissues, and correlated with decreased expression levels of H3K27me3 in the same tissues [59].

The above data highlight that the prognostic role of H3K27me3 may vary between different cancer types. This is observed also with the H3K36me3 mark, as described further below. One possible explanation is that the function of histone marks may be cell context dependent in that, based on the cellular context, H3K27me3 may silence different downstream gene targets (i.e. more oncogenes rather than tumor suppressors). Furthermore, each mark may be “read” and/or opposed by other histone marks, and participate in different molecular complexes that induce divergent downstream pathways in different cancer types. Another important point highlighted by these studies is that EZH2 and H3K27me3 levels do not always correlate in certain cancer types. This implies that EZH2 may have a different substrate(s) other than H3K27 and may be driving oncogenesis through non-H3K27me3-mediated processes in these cancer types. It has also been postulated that higher levels of EZH2 may disrupt the PRC2 complex necessary for EZH2-mediated H3K27me3, and that EZH2 may then bind to different interacting proteins, whereby H3K27 is no longer its substrate [59].

The above studies underscore the important role of H3K27me3 as an independent prognostic factor in different cancer types. H3K27me3-based prognosis is cancer type specific, where high levels impact poor prognosis in colon cancer, HCC, and ESCC, while an inverse pattern is observed in breast and lung cancers. The potential factors that influence this differential correlation of H3K27me3 and cancer prognosis merits further investigation.

### H3K36me3

H3K36, which refers to lysine 36 from the N-terminus of histone H3, is associated with actively transcribed genes, especially when it undergoes tri-methylation (H3K36me3) [60]. H3K36me3 is thought to function in DNA mismatch repair, modulation of chromatin structure, and stem cell regulation [60]. SETD2 is the only methyltransferase thought to tri-methylate H3K36 in the literature [66]. Interestingly, similar to the prognostic significance of H3K27me3, H3K36me3 expression levels have been correlated with both better or worse survival in different cancer types. Lien et al. demonstrated that in HCC, H3K36me3 positivity on immunostaining is not only a predictor of high tumor grade and stage, but is also a contributor to tumor recurrence and poor survival [60]. In contrast, Ho et al. discovered that even when adjusting for age and a validated prognostic scoring, loss of H3K36me3 expression was associated with higher risk of renal cell carcinoma-specific death and progression after nephrectomy in clear cell renal cell carcinoma patients [61]. These seemingly contradicting results underscore the fact that the function of specific histone marks, as well as chromatin modifiers, may be cell context specific and may differ in each different cancer type, as described above regarding the H3K27me3 mark.

### H3K9 methylation

Chromatin modifications are extensively involved in gene regulation, with modification of lysine residues playing an important role in disease biology. H3K9 represents lysine 9 on histone H3. It is modified through the action of a number of PKMTs which add mono-, di-, or tri-methyl groups to the lysine. While H3K9me1 is associated with open chromatin and active gene expression, H3K9me2 and H3K9me3 are involved in repression or silencing of gene expression. This section focuses on the role of H3K9me3, a well studied methylation mark, in cancer prognosis.

In 52 colorectal cancer tissues obtained from stage II and III patients, Yokoyama et al. reported elevated H3K9me3 levels in the invasive region of tumor tissues, and the intensity of H3K9me3 staining correlated positively with lymph node metastasis [62]. In a study by Song et al. on 408 NSCLC tissues, a positive correlation between H3K9me3 and tumor recurrence was observed [64]. Moreover, in another study conducted by Zhou et al. on 135 pairs of ESCC and corresponding non-tumor tissue samples, high levels of H3K9me3 were associated with poor prognosis [65]. In addition, Park et al. studied 261 gastric adenocarcinoma samples for their patterns of H3K9me3, as well as H3K9ac, H4K16ac and H4K20me3 [63]. Higher H3K9me3 levels significantly correlated with advanced tumor stage, lymphovascular invasion, cancer recurrence, and poor overall survival, and H3K9me3 was found to be an independent prognostic factor for gastric adenocarcinoma patients [63].

The above studies support that high H3K9me3 levels are associated with cancer progression, distant metastasis, and overall lower patient survival in colon cancer, NSCLC, ESCC, and gastric cancer, and suggest that H3K9me3 may be an independent prognostic factor in these cancer types.

## PKMT inhibitors in clinical trials

The investigation of PKMTs in various cancer types has revealed their importance as key mediators of oncogenesis and cancer progression, and has highlighted the promise of some of these as cancer therapeutic targets. In this section, we discuss updates on PKMT inhibitors that have entered clinical trials for solid tumors.

In a recent review, Copeland et al. reported the existence of small molecule inhibitors targeting 11 PKMTs with mechanisms that include S-adenosyl-L-methionine (SAM) competition, peptide-site binding inhibition, allosteric inhibition, and complex disruption [67]. Table 3 lists all available PKMT inhibitors, their mechanisms of action, and clinical trial information when available [67]. Of the PKMTs discussed in this manuscript, small molecule inhibitors exist for EHMT2, EZH2, SETD7, SMYD2, and SMYD3 [68]. EZH2 and Disruptor of Telomeric Silencing 1-like (DOT1L) inhibitors have already entered clinical trials [69]. Currently, there are five EZH2 targeting inhibitors that have entered clinical trials with an indication for solid tumors. There is currently one DOT1L targeting inhibitor that has entered clinical trials for hematologic malignancies [69]. These inhibitors are briefly discussed below. Given the scope of this review, this discussion mainly focuses on PKMT inhibitors that have been tested in solid tumors rather than blood malignancies. Inhibitors of protein demethylases are not included in this review.

**Table 3:** PKMT inhibitors in various stages of clinical development

**EZH2**		
**Compound name**	**Mechanism of action**	**Clinical trial information (****clinicaltrials.gov****)**
Tazemetostat (EPZ-6438, Epizyme)	SAM-competitive	**Hematologic malignancies:** -**Tazemetostat monotherapy:** NCT03009344, NCT03456726, NCT02220842 (completed) -**Tazemetostat in combination:** NCT04224493 (Tazemetostat + Lenalidomide/Rituximab) **Hematologic + solid tumors:** -**Tazemetostat monotherapy:** NCT02875548, NCT03010982 (completed), NCT01897571, NCT03213665, NCT03028103, NCT03155620 **Solid tumors:** -**Tazemetostat monotherapy:** NCT02601950, NCT02860286 (completed), NCT04241835, NCT02601937 -**Tazemetostat in combination:** NCT04179864 (Tazemetostat + Abiraterone/Enzalutamide), NCT04204941 (Tazemetostat + Doxorubicin), NCT03854474 (Tazemetostat + Pembrolizumab)
GSK2816126 (GlaxoSmithKline)	SAM-competitive inhibition (PRC2-specific inhibitor)	1 terminated trial
CPI-1205 (Constellation Pharmaceuticals)	SAM-competitive inhibition (PRC2-specific inhibitor)	**Hematologic malignancies**: **-CPI-1205 monotherapy:** NCT02395601 (completed) **Solid tumors**: -**CPI-1205 in combination:** NCT03480646 (CPI-1205 + Abiraterone/Enzalutamide), NCT03525795 (CPI-1205 + Ipilimumab)
MAK683 (Novartis)	PRC2 complex disruptor (binds to EED)	**Hematologic malignancies:** **-MAK683 monotherapy:** NCT02900651
**PRC2 Complex**		
**Compound name**	**Mechanism of action**	**Clinical trial information (****clinicaltrials.gov****)**
SAH-EZH2 (Calbiochem)	Disruption of PRC2 subunit interactions	Preclinical use
A-395	Disruption of PRC2 subunit interactions	Preclinical use
**EZH1/EZH2**		
**Compound name**	**Mechanism of action**	**Clinical trial information (****clinicaltrials.gov****)**
Valemetostat (DS-3201b, Daiichi Sankyo)	EZH1/EZH2 inhibitor with equal affinity via SAM-competitive inhibition	**Hematologic malignancies:** **-Valemetostat monotherapy:** NCT03110354, NCT04102150, NCT02732275 **Solid tumors:** -**Valemetostat in combination:** NCT03879798 (Valemetostat + Irinotecan), NCT04388852 (Valemetostat + Ipilimumab) **Hepatic impairment**: **-Valemetostat monotherapy:** NCT04276662
899145	SAM-competitive inhibition	Preclinical use
**DOT1L**		
**Compound name**	**Mechanism of action**	**Clinical trial information (****clinicaltrials.gov****)**
Pinometostat (EPZ-5676, Epizyme)	SAM-competitive inhibition	**Hematologic malignancies:** **-Pinometostat in combination:** NCT03701295 (Pinometostat + Azacitidine), NCT03724084 (Pinometostat + Cytarabine/Daunorubicin/Daunorubicin Hydrochloride)
**Menin-MLL**		
**Compound name**	**Mechanism of action**	**Clinical trial information (****clinicaltrials.gov****)**
MI-503	Disruption of MLL complex subunit interactions	Preclinical use
**EHMT1/EHMT2**		
**Compound name**	**Mechanism of action**	**Clinical trial information (****clinicaltrials.gov****)**
UNC0642	Peptide-competitive inhibitor	Preclinical use
**SUV420H1/2**		
**Compound name**	**Mechanism of action**	**Clinical trial information (****clinicaltrials.gov****)**
A-196	Peptide-competitive inhibitor	Preclinical use
**SMYD2**		
**Compound name**	**Mechanism of action**	**Clinical trial information (****clinicaltrials.gov****)**
EPZ033294	Peptide-competitive inhibitor	Preclinical use
**SMYD3**		
**Compound name**	**Mechanism of action**	**Clinical trial information (****clinicaltrials.gov****)**
EPZ031686	Peptide-competitive inhibitor	Preclinical use
**SETD7**		
**Compound name**	**Mechanism of action**	**Clinical trial information (****clinicaltrials.gov****)**
(*R*)-PFI-2	Peptide-competitive inhibitor	Preclinical use
**SETD8**		
**Compound name**	**Mechanism of action**	**Clinical trial information (****clinicaltrials.gov****)**
UNC0379	Peptide-competitive inhibitor	Preclinical use

### EZH2 inhibitors that function through SAM competition

Tazemetostat (EPZ-6438) (Epizyme) was the first EZH2 inhibitor to enter clinical trials for solid malignancies [69]. Its mechanism of action is via SAM-competitive inhibition of EZH2. Tazemetostat has been investigated in a phase 1/2 study (phase 1 complete, phase 2 ongoing) in both advanced solid tumors as well as hematologic malignancies (NCT01897571) [70]. Results of the phase 1 study showed a favorable safety profile and meaningful antitumor activity with durable objective responses in 38% of patients with refractory B-cell non-Hodgkin lymphoma and clinical benefit in 38% of patients with *INI1*- and *SMARCA4*-negative solid tumors, such as epithelioid sarcomas [70]. Tazemetostat received FDA approval in January of 2020 for the treatment of adults and pediatric patients aged 16 years and older with metastatic or locally advanced epithelioid sarcoma not eligible for complete resection. Furthermore, there are currently 14 active phase 1/2 clinical trials involving tazemetostat in multiple solid tumor malignancies, such as bladder, breast, colorectal, NSCLC, melanoma, prostate, urothelial, renal cell carcinoma, endometrial and ovarian cancer [69]. Among these studies, tazemetostat is being investigated in combination with pembrolizumab (PD-1 inhibitor) in a phase 1/2 study of patients with locally advanced and metastatic urothelial carcinoma and bladder carcinoma (NCT03854474), as well as in a phase Ib study in combination with atezolizumab (PD-L1 inhibitor) in relapsed or refractory diffuse large B-cell lymphoma (NCT02220842). Finally, a phase 1b trial is ongoing, investigating tazemetostat, abiraterone (androgen suppressor) plus prednisone (corticosteroid), or enzalutamide (androgen receptor antagonist) in metastatic prostate cancer patients (NCT04179864). Additionally, a phase 2 trial investigating tazemetostat in relapsed/refractory malignant mesothelioma with BAP1 loss of function has been completed, and preliminary results demonstrated that tazemetostat monotherapy showed promising antitumor activity with favorable safety in these patients (NCT02860286) [69, 71].

GSK2816126 (Glaxo Smith Klein) is a PRC2-specific inhibitor of EZH2 that acts via SAM-competitive inhibition [67]. GSK2816126 was being investigated in a phase 1 trial for solid tumors and other hematologic malignancies, but was terminated early as the maximal dose and schedule attained with GSK2816126 showed insufficient clinical activity to justify further investigation (NCT02082977) [69].

CPI-1205 (Constellation Pharmaceuticals) is a PRC2-specific inhibitor of EZH2 via SAM-competitive inhibition [67]. There are currently two ongoing clinical trials with CPI-1205, both of which involve solid tumors [69]. One trial is a phase 1/2 study investigating CPI-1205 plus ipilimumab in advanced solid tumors (NCT03525795) [69]. The other trial is a phase 1/2 study investigating CPI-1205 in combination with enzalutamide or abiraterone/prednisone in patients with metastatic castration-resistant prostate cancer (NCT03480646) [69].

DS-3201b (Daiichi Sankyo) is unique compared to the other currently available PRC2-specific EZH2 inhibitors, as it inhibits both EZH1 and EZH2 with equal affinity via SAM-competitive inhibition [67]. DS-3201b is currently being studied in six clinical trials, two of which have a solid tumor indication [69]. One is a phase 1/2 trial investigating DS-3201b in combination with irinotecan in patients with recurrent small cell lung cancer (NCT03879798). The other is a phase 1b trial investigating DS-3201b in combination with ipilimumab (CTLA-4 inhibitor) in metastatic prostate, urothelial, or renal cell carcinoma (NCT04388852) [69].

### EZH2 inhibitors that disrupt the PRC2 complex

MAK683 (Novartis) is a PRC2 complex disruptor that binds to the Embryonic Ectoderm Development protein (EED) [67]. It is currently being studied in a phase 1/2 clinical trial indicated for advanced solid tumor malignancies and diffuse large B-cell lymphoma (NCT02900651) [69].

### DOT1L inhibitor that functions through SAM competition

Pinometostat (EPZ-5676, Epizyme) is a DOT1L disruptor via SAM competitive inhibition [67]. It is currently being investigated in two active phase 1b/2 clinical trials studying acute myeloid leukemias and leukemia cutis (NCT03701295, NCT03724084) [69].

## Conclusion

PKMTs constitute a family of enzymes that are known to “write” mono-, di- or tri-methylation marks on specific lysine residues of histone and non-histone substrates, leading to either activation or repression of specific transcriptional programs and protein functions within the cell. An abundance of preclinical studies over the past 30 years have unveiled the importance of many of these epigenetic writers in oncogenesis and cancer progression. The Cancer Genome Atlas has also revealed multiple and, in some cases, recurrent genetic and expression alterations in some of these enzymes in multiple cancer types, underscoring their significance in cancer biology.

Given the known discrepancy between mRNA and protein levels in cancer cells, in this review, we attempted to summarize clinicopathologic associations of studies that have evaluated the protein expression levels of specific PKMTs in different cancer types using immunohistochemistry in clinically annotated tissue samples. While immunohistochemistry bares inherent shortcomings, such as variability in technique between different laboratories, operator-dependent bias in the interpretation of staining results, semiquantitative analysis of results, and limitations related to the quality of the antibodies used, we included studies that fulfilled the majority of the REMARK criteria and analyzed sufficient number of tumor samples to attain statistical significance. Furthermore, immunohistochemistry enables the distinction between cancer and stroma/immune cells, thus ascertaining that the associations derived are cancer-cell specific. Regardless, as proteogenomic databases are being developed in multiple cancer types, it will be important to conduct similar analyses using these databases which will provide the opportunity for more accurate and quantitative assessments of such clinicopathologic associations.

Our review highlights that a number of PKMTs are associated with diverse clinicopathologic features, such as clinical stage or histologic grade, and influence survival outcome independently of known prognostic factors, underscoring their importance as potential drivers in a variety of cancer types. However, overexpression of any PKMT, even if this is associated with statistically significant correlations with important clinicopathologic cancer features, does not prove causality and necessity for oncogenic processes, and rigorous preclinical investigation is needed to this purpose.

An interesting and important point that is highlighted through this review is that higher expression of a PKMT does not necessarily correlate with higher expression of its known histone mark, with a major example being EZH2 and H3K27me3 expression in breast cancer [57]. In these studies, higher expression levels of EZH2 were associated with worse survival, while higher H3K27me3 levels were associated with improved survival. Such contrasting results underline the fact that there may be other histone or non-histone substrates through which a PKMT may exert oncogenic effects, or that enzymatically inactive PKMTs may activate oncogenic mechanisms in certain cancer types. Another important point that this review highlights is that the function of PKMTs and/or histone marks seem to be cell context specific, and may vary (or be seemingly contrasting) among different cancer types. This is well exemplified with the contrasting effects of H3K27me3 on the survival of breast and hepatocellular carcinoma patients, as well as H3K36me3 on the survival of hepatocellular and renal cell carcinoma patients. This notion is crucial for clinical translation and it suggests that biologic mechanisms of PKMTs and histone marks in a specific cancer type should avoid being extrapolated to other cancer types.

Of all PKMTs, EZH2 has been the most studied and EZH2 inhibitors have already entered clinical trials in multiple cancer types. It is interesting to note that EZH2 has been implicated as an oncogene in multiple cancer types, raising the question of whether it can be considered a universal anticancer drug target. Indeed, numerous studies on EZH2 have highlighted its role in cell survival and proliferation, epithelial to mesenchymal transition and invasion, and immune evasion [72], underscoring its function as a master regulator of multiple hallmarks of cancer. Ongoing trials investigating EZH2 inhibitors in multiple cancer types as described in this review will provide insight to this question, however, accurate biomarkers of response to EZH2 inhibition will be of paramount importance in deciphering this question.

Multiple preclinical studies have elucidated mechanisms of function of multiple other PKMTs, and small molecule compounds are currently under development to target some these enzymes. An important aspect to be considered in this endeavor is to ensure that the targeted PKMTs are either absent or minimally expressed in normal vital organs, in order to decrease potential off-target toxicities. It will also be important to identify predictive biomarkers of response to these drugs, which would entail assays that allow determination of overexpression of the specific target. Furthermore, deciphering specific mechanisms of action of PKMTs in different cancer types will be crucial for the identification of appropriate biomarkers of pharmacodynamic efficacy, as well as mechanisms of drug resistance.

## Explanation of staging and statistical terms

### TNM staging

This is the most widely used cancer staging system [73]. T (tumor) refers to the size and extent of the primary tumor, N (node) refers to the number of lymph nodes that have been invaded by cancer, M (metastasis) refers to whether the cancer has spread to other organs of the body. TNM staging was created by the American Joint Committee on Cancer (AJCC) and the International Union Against Cancer (UICC).

### Prostate-specific antigen (PSA)

Prostate-specific antigen is a substance made mostly by the prostate that can be found in increased amounts in the blood of men with prostate cancer [74]. Due to these findings, a PSA test is often performed to screen men for prostate cancer.

### FIGO staging

FIGO(International Federation of Gynecology and Obstetrics) staging is a system commonly used in conjunction with TNM staging for female genital cancers such as vulvar, vaginal, cervical, uterine, ovarian, primary peritoneal carcinoma, fallopian tube, and gestational trophoblastic tumors [75]. FIGO staging consists of 4 stages; Stage 0: in situ carcinoma; Stage I: localized, confined to organ of origin; Stages II-IV: extension beyond organ of origin.

### Univariate vs multivariate analysis

Univariate, bivariate, and multivariate analyses are three tools used in statistical analysis. Univariate analysis is the analysis of data using one variable, bivariate analysis is the analysis of data using two variables, and multivariate analysis is the analysis of data using three or more variables. For example, if one is analyzing a colon cancer cohort and variables include stage, lymph node infiltration, and metastasis, univariate analysis could be employed to analyze the cohort strictly on one variable: stage, lymph node infiltration, or metastasis. However, the other two variables would be ignored. On the other hand, multivariate analysis could be used to analyze the colon cancer cohort using all three variables at the same time.

## Data Availability

Not applicable.

## Abbreviations

PKMT: Protein lysine methyltransferase
EZH2: Enhancer of Zeste Homolog 2
TNBC: Triple negative breast cancer
HER2: Human Epidermal Growth Factor Receptor 2
ER/PR: Estrogen receptor/progesterone receptor
SETD1A: SET Domain Containing 1A
SETD7: SET Domain Containing Lysine Methyltransferase 7
TNM: Tumor, node, metastasis
NSCLC: Non-small cell lung cancer
EGFR: Epidermal Growth Factor Receptor
EHMT1: Euchromatic Histone Lysine Methyltransferase 1
EHMT2: Euchromatic Histone Lysine Methyltransferase 2
SETDB1: SET Domain Bifurcated 1
SMYD3: SET and MYND Domain Containing 3
PSA: Prostate specific antigen
NSD2: Nuclear Receptor Binding SET Domain Protein 2
HCC: Hepatocellular carcinoma
SMYD2: SET and MYND Domain Containing 2
SCCHN: Squamous cell carcinoma of the head and neck
NSD3: Nuclear Receptor Binding SET Domain Protein 3
SAM: S-adenosyl-L-methionine
PRC2: Polycomb Repressive Complex 2
EED: Embryonic Ectoderm Development Protein
DOT1L: Disruptor of Telomeric Silencing 1-Like
FIGO: International Federation of Gynecology and Obstetrics
HPV: Human papillomavirus
